# Efficacy of PowerBall Versus Mulligan Mobilization With Movement on Pain and Function in Patients With Lateral Epicondylitis: A Randomized Clinical Trial

**DOI:** 10.7759/cureus.56444

**Published:** 2024-03-19

**Authors:** Shivani R Uttamchandani, Pratik Phansopkar

**Affiliations:** 1 Musculoskeletal Physiotherapy, Ravi Nair Physiotherapy College, Datta Meghe Institute of Higher Education and Research, Wardha, IND

**Keywords:** mulligan mobilization with movement, grip strength, pain and function, extensor carpi radialis brevis muscle, lateral epicondylitis, “powerball device”

## Abstract

Background

Lateral epicondylitis (LE), sometimes referred to as tennis elbow or lateral elbow tendinopathy (LET), is one of the most common repetitive stress disorders in the elbow joint. Often, this involves the attachment of the extensor carpi radialis brevis muscle. This study's primary focus is on treating people with LE, a condition that causes repetitive movements of the upper extremities. There is currently no research on how PowerBall gadget workouts affect the function and pain of individuals with lateral epicondylitis. Exercises using the "PowerBall device," which applies both intrinsic and extrinsic pressure to the wrist, elbow, and shoulder muscles, are thought to be beneficial forms of resistance training. It has been shown that there are improvements in strength, function, range of motion (ROM), discomfort, and quality of life (QOL). On the other side, it has been demonstrated that LE patients have reduced discomfort while using Mulligan Mobilization with Movement (MMWM).

Methods

The 50 patients with LE were split into two groups for the single-blinded, randomized clinical study after baseline assessment and randomization: Group A was the intervention group, and Group B was the conventional group. The "PowerBall device" exercise was provided to participants in Group A, and MMWM was given to those in Group B. Both groups can benefit from basic workouts and ultrasonography by following the prescribed routine. Quantification of pain, function, grip strength, and range of motion was done at the start and finish of therapy using the Visual Analogue Scale (VAS), Patient Rated Tennis Elbow Evaluation (PRTEE), portable dynamometer, and goniometer.

Results

After therapy, both groups showed considerable improvement (p<0.05). Both descriptive and inferential statistics were employed in the data analysis. Numerous statistical tests were employed, such as the student's paired and unpaired t-test and the chi-square test. From a statistical and clinical perspective, Group A's outcomes were more significant. On the visual analog scale, there was a decrease in pain intensity for wrist and elbow mobility at rest (p<0.0003), activity (p<0.003), PRTEE (p<0.001), grip strength (p<0.03), and range of motion (p<0.01). Both groups' assessments after rehabilitation indicated increases in pain and function; however, Group A (0.03) benefited more and saw early success with the PowerBall device.

Conclusion

Findings show that a three-week program incorporating resistance training exercises mediated by a "PowerBall device" enhances upper limb performance beyond traditional exercise treatment and increases grip strength, wrist extension strength, internal and external rotator concentric and eccentric strength. The findings and observations indicate that both groups have significantly improved.

## Introduction

One of the most common arm ailments is lateral epicondylitis (LE), sometimes referred to as tennis elbow or lateral elbow tendinopathy (LET) [1]. The lawn tennis arm was initially described by Morris in 1882 [2]. Tendinopathies, a set of pathologies that make up a continuum of diseases, are tendon abnormalities caused by prolonged overuse. This disorder affects the lateral section of the forearm's extensor tendons and is most frequently discovered in middle-aged individuals, with an incidence range of 40-60 years [3]. According to demographic surveys, incidence among people aged 30 to 64 ranges from 1-3%, with the global population peaking between 45 and 54 [4]. There is no gender difference in the incidence or prevalence of LE, despite the disorder affecting 1-4% of the Indian population who repeatedly move their upper extremities in activities like using a computer, carrying heavy objects, and being subjected to repeated vibration [5].

The elbow's lateral epicondyle, an extensor muscle origin in particular, is affected by LE, a common musculoskeletal disorder that results from repeated microtoroids to the upper extremity [6]. In particular, resistive wrist and middle finger extension can cause sensitivity, discomfort in the lateral epicondyle, and chronic pain syndrome if treatment is not received. It is simple to diagnose LE with a clinical examination. Activities of daily living (ADL) may become severely restricted, and grip strength may also deteriorate [7]. Overuse is a significant factor, as it happens more frequently in the dominant arm. According to observations, the dominant arms of around 75% of the population are impacted. During clinical and surgical procedures, the muscles of the extensor digitorum communis (EDC), extensor carpi radialis longus (ECRL), and extensor carpi ulnaris (ECU) have the potential to draw in. Most often, the extensor carpi radialis brevis (ECRB) is the muscle that pulls in [2].

Usually, a work- or sport-related ailment, wrist clutching, and lengthy, abrupt, uniform, repetitive eccentric contractions are the culprits. The term "tennis elbow" is misleading because, in 40-50% of situations, participating in racquet sports raises the chance of having this illness, and it only manifests in 5-10% of tennis players [8]. It is frequently observed as a result of the upper limb's reliance on nearby muscles, ligaments, and capsules to codirect movement [9]. Contrary to its name, this chronic sports-related illness is common in baseball, swimming, squash, and throwing activities, among other sports. There is a higher incidence among persons who play racquet sports and work in high-risk occupations that frequently involve gripping, wrist extension, and loading activities such as carrying heavy objects. When high loading is combined with personal risk factors, including age, occupation, hobbies, or past injuries, degenerative tendinosis develops from normal tendons [3].

There is currently no ideal course of treatment for LE, even though its clinical signs are obvious and simple to identify. Evidence suggests that the moderate and long-term effects of corticosteroid injections are actively predicted to be poorer than those of controls [10]. Being the preferred course of treatment for LE, many medical professionals support a conservative approach. In most cases, people with LE are given physiotherapy as a conservative treatment. The LE exercise regimen is the most widely used in physical therapy rehabilitation. Exercise regimens often include both exercises done in a clinical environment and at-home workouts [1]. Education and workplace ergonomic guidance are also frequently given. Several treatments have been shown to help people with lateral epicondylitis. These include myofascial release, exercise therapy, manipulations and mobilizations, taping, shock wave treatment, therapeutic ultrasound, phonophoresis, iontophoresis, and many more. These treatments can help reduce pain and improve function [10,11].

According to recent studies, vibrating devices like the PowerBall gadget have a greater effect on people with tennis elbow injuries than dumbbell exercise does on muscular strength, discomfort, range of motion, and health quality. Exercises using the "PowerBall device" are thought to be effective resistance training during the chronic period because the muscles of the wrist, elbow, and shoulder, apply both internal and external pressure [9]. Muscle strength may have grown over this period because of neurovascularization, the creation of new blood vessels during the eccentric phase of training that enhances blood flow and speeds up tissue repair [12]. 

The manual therapy technique known as Mulligan Mobilization with Movement (MMWM) entails a prolonged lateral glide and a simultaneous physiological movement of the elbow joint. The objective of MMWM is to mobilize the joint while it is moving spontaneously, in contrast to standard mobilization procedures, which perform mobilization in a static position. Studies show that MMWM is beneficial for pain reduction and restoring the elbow joints' functional capacity in LE [11]. MMWM may have a neurophysiological basis since it stimulates soft tissues with compression and a tactile reaction simultaneously [13]. It could be able to restore the spinal cord circuitry by permitting an individual to do repetitive, painless motions. By doing so, it might be possible to disable maladaptive spinal cord circuitry and return nociception and motor neuron pool excitation to normal [14]. This offers an alternative to Mulligan's theories, which hold that the malfunction in lateral epicondylitis is caused by a positional fault or obstruction of the joint. Mulligan anticipated that repositioning the joint in conjunction with joint motion would correct the positional fault of the joint [15].

This protocol was published on Protocol Exchange, an accessible database of community-contributed protocols sponsored by Nature Portfolio, on May 28, 2021 (https://www.researchsquare.com/article/pex-1495/latest).

## Materials and methods

Participants

**Figure 1:**
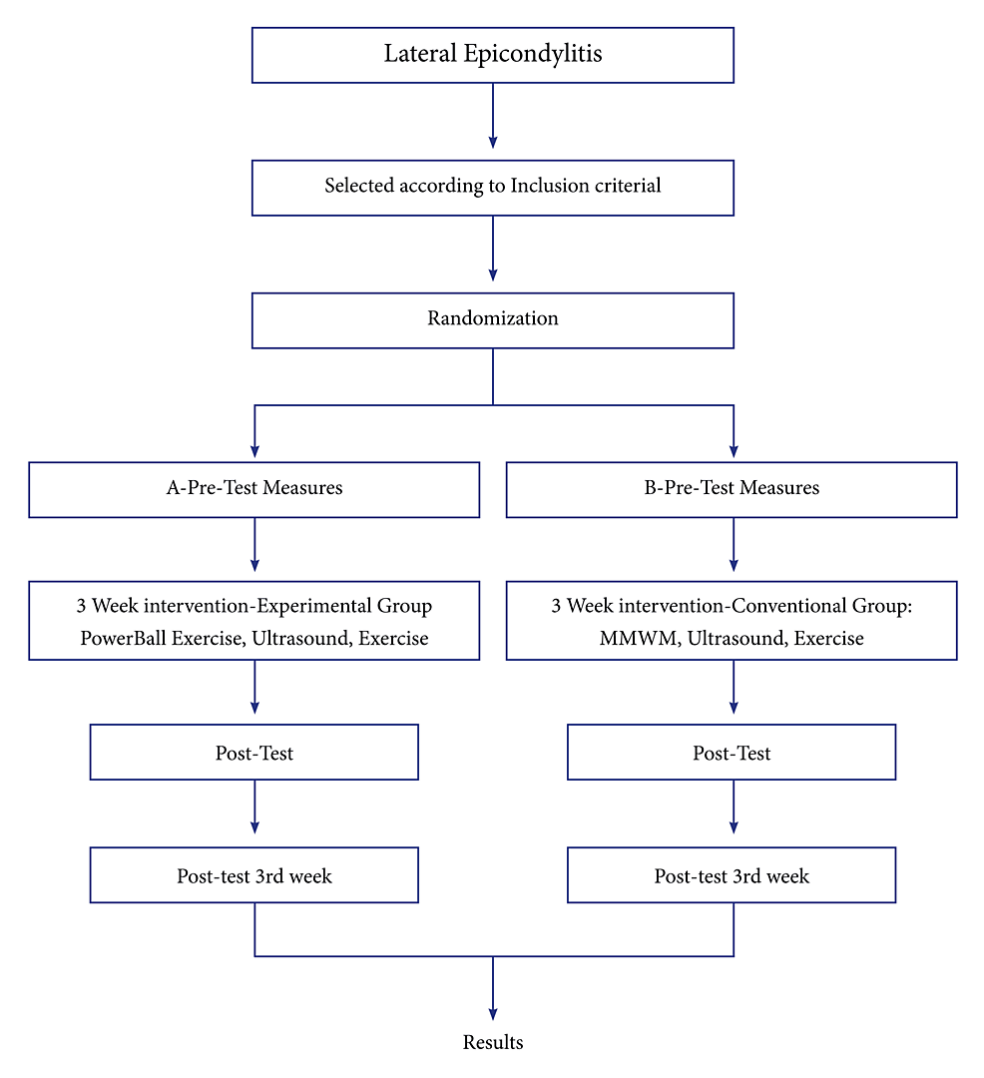
Flowchart of the study MMWM: Mulligan Mobilization with Movement

Participants in the research were those between the ages of 20 and 50 who experienced elbow discomfort for more than two weeks [3]. There were 50 patients in the study, 25 in each of Groups A and B. Between June 2021 and June 2022, the recruitment process was represented as a straight line. The inclusion criteria were that every participant had experienced symptoms for more than two weeks, their age should range from 20 to 50, their ECR tendon should be tender on palpation, the physical examination used to confirm the diagnosis should be carried out by Mill's test, and the study participant must be able to fulfill the outcome measures and be willing to engage in the research. Patients with elbow fractures, any prior surgical experience at the elbow joint, any prior instability of the elbow joint, any underlying malformations (excessive carrying angle, hyperextended elbow), or any history of recent trauma or neurological illness were excluded. Each participant gave their written, informed consent. Baseline data is available for a total of 50 randomized people. The method used in the research is depicted in a flowchart in Figure 1.

Study design

In Sawangi (Meghe), Wardha, India, a randomized clinical study was carried out at the Acharya Vinoba Bhave Rural Hospital and the Musculoskeletal-Physiotherapy Outpatient Department of the Ravi Nair Physiotherapy College. Prior to starting the study, the research protocol was published on protocol exchange. Using the sequentially numbered opaque sealed envelope (SNOSE) approach, the included persons who had been diagnosed with LE were split into two groups: Group A (PowerBall exercise group) and Group B (Mulligan Mobilization with Movement (MMWM)). A postgraduate resident in physiotherapy with an equivalent level of expertise evaluated the results before the start of the trial and just after it was over. This individual was told about the experiment and blinded to the intervention. The study's enrollment, intervention, and assessment procedures all met the protocol's requirements, which included a suggestion to conduct intervention trials. Before being accepted, participants were told of the study's objectives and procedures and granted consent on a written patient permission form.

Procedure

**Figure 2:**
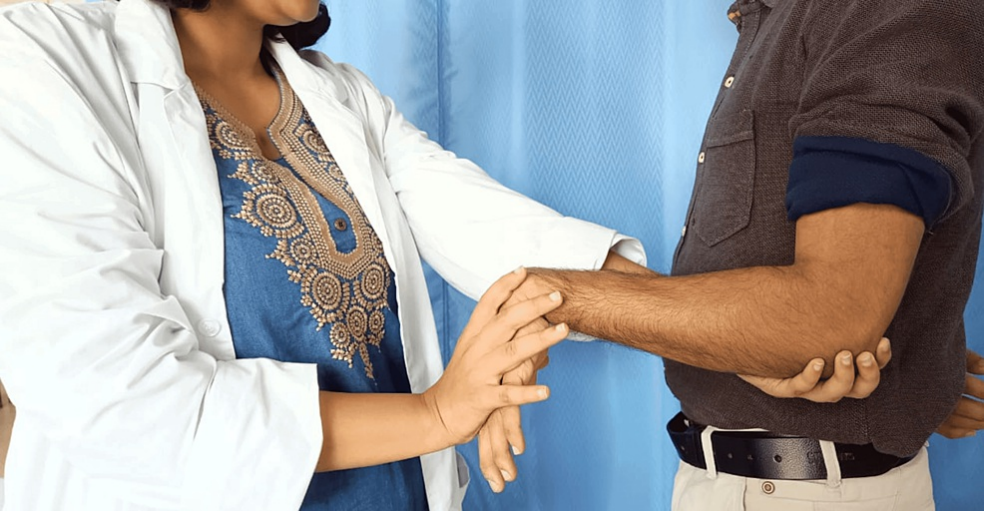
Mill’s test performed by therapist to diagnose lateral epicondylitis [17]

At baseline, the socio-demographic information for the participant's name, age, sex, occupation, and address was gathered and recorded. In addition to measuring with a dynamometer, the degree of disability and function were evaluated using the Patient Rated Tennis Elbow Evaluation (PRTEE), and the pain was measured with a Visual Analogue Scale (VAS). Before starting the therapy and again after the third week, recordings were made. Based on the results of Mill's test, as shown in Figure 2, patients were diagnosed. The patient sat comfortably for the evaluation of their pain-free grip strength with their arm at their side [16]. The patient was advised to exert their greatest amount of pressure for three to five seconds. To prevent the potential impact of exhaustion, a pause of around 15 seconds was observed between each repetition of the average of the three trials that were provided.

Intervention

**Figure 3:**
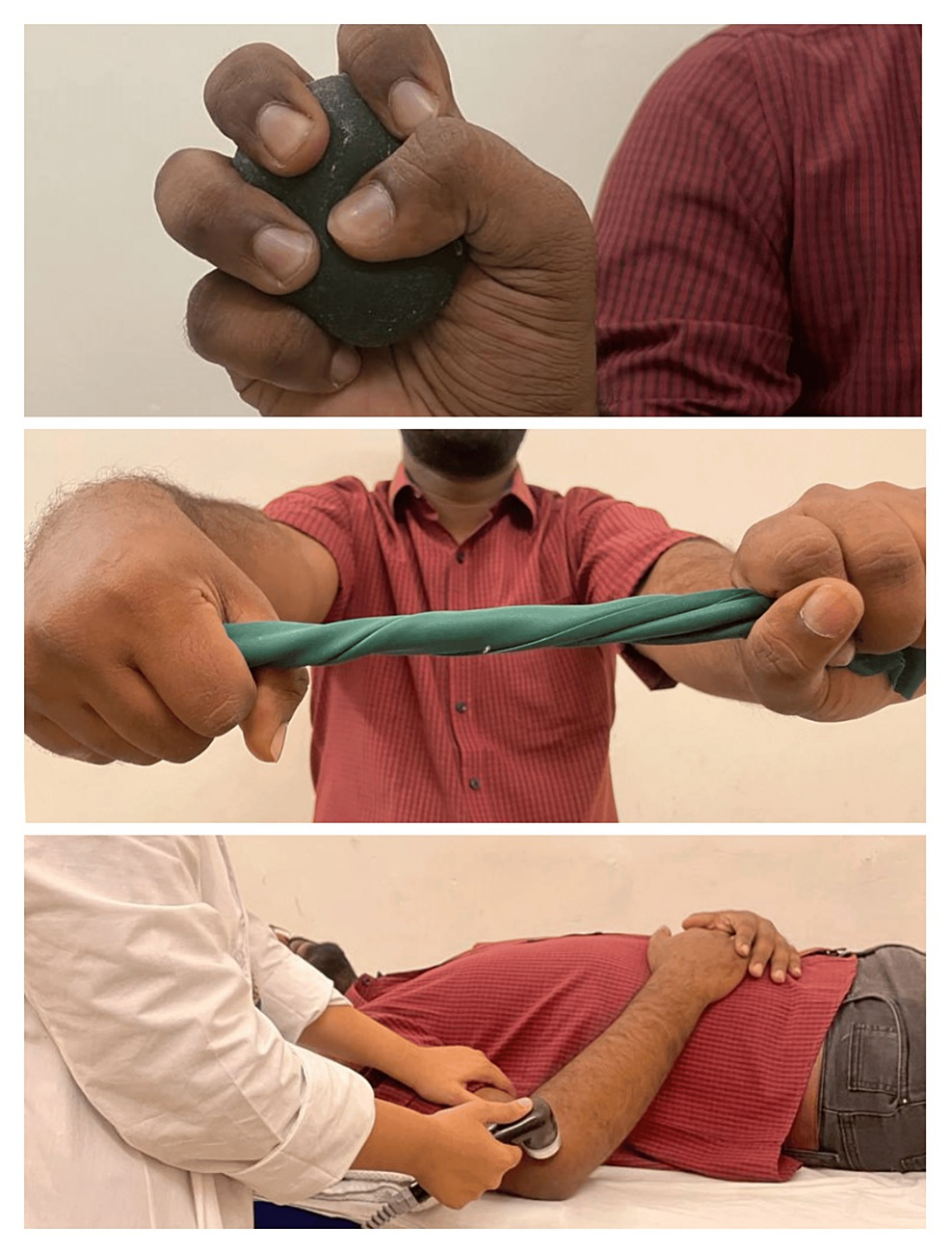
Shows Ball squeezing, twisting rubber bands, and ultrasound

**Figure 4:**
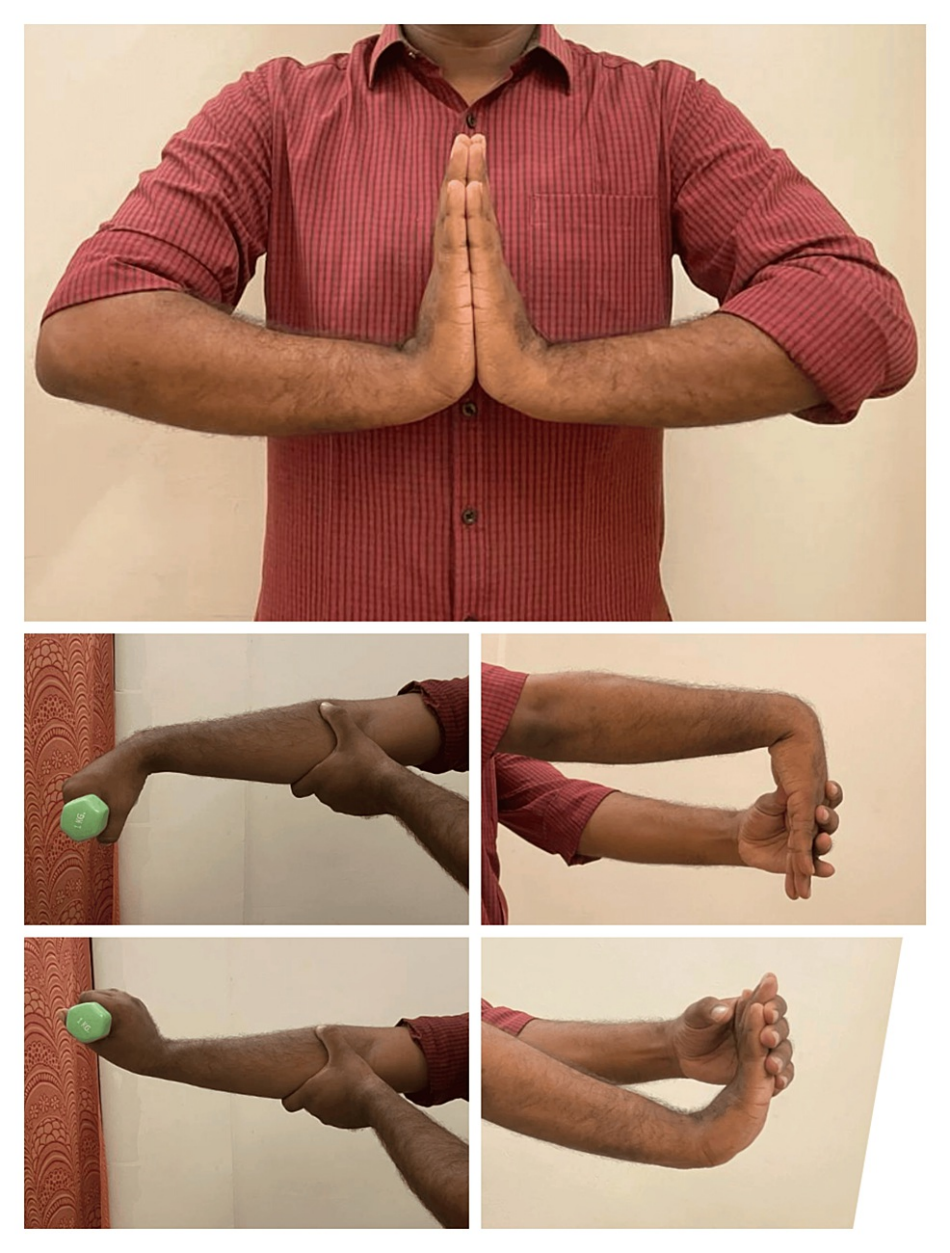
Shows forearm strengthening exercises with a dumbbell, forearm stretching exercises, and a namaste stretch

Groups A and B were created at random from 50 patients with LE. Patients in Group A received PowerBall device exercises using a PowerBall, and those in Group B received MMWM. For the next three weeks, the participants underwent the chosen treatment for five sessions a week. Before doing Powerball exercises, or MMWM, both groups received ultrasounds and exercises. Patients sat on the chair, keeping their shoulders in a neutral position with the fully supported elbow at 90°. Ultrasound was performed at the tenderness point in pulsed mode at 1 w/cm2 for 10 minutes. Firm fist clenching as well as wrist flexor and extensor resistance workouts were among the exercises (strength training for muscles using DeLorme’s principle), radioulnar movements, and stretching of the wrist for at least 30 seconds. Every exercise was performed for three sets 10 times, as shown in Figure 3 and Figure 4. 

Group A (Experimental Group)

**Figure 5:**
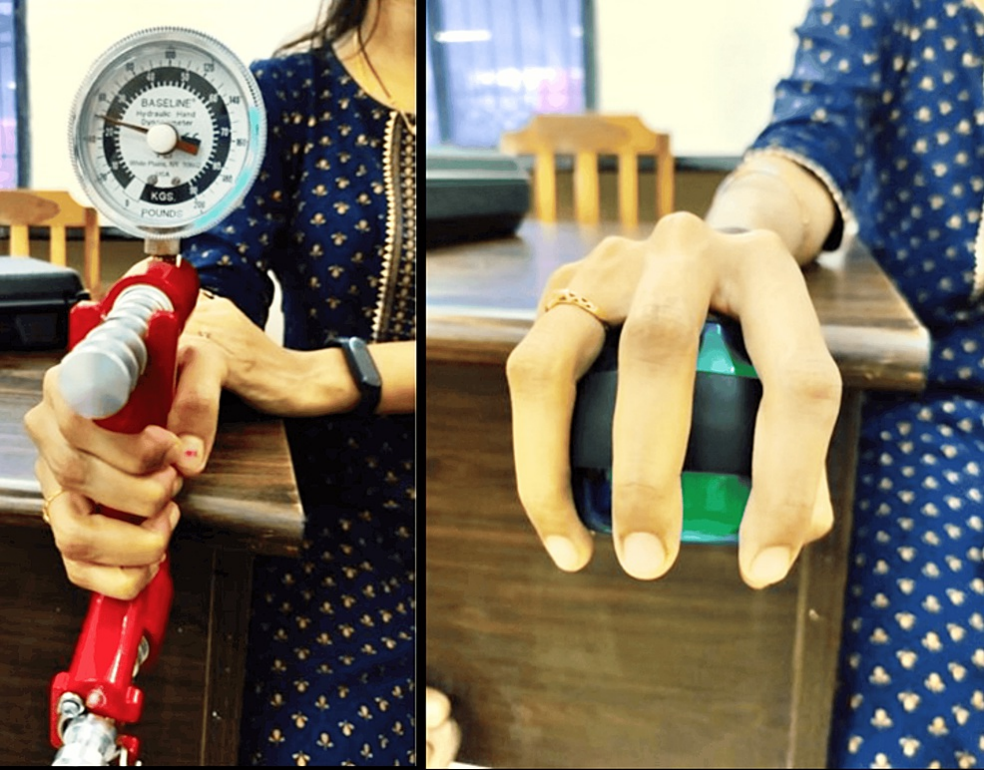
Showing the grip strength in a female aged 29 years

For the next three weeks, consistently, there were five sessions each week of PowerBall training in this group. Each session lasted somewhere between 20 and 64 minutes. The "PowerBall gadget" varied its intensity from 2000 up to 10,000 gallons per minute, depending on the participant's capacity. Following ultrasound and strength training, PowerBall training was given to Group A. The participant used a PowerBall while seated, holding the ball in their hand and laying their arm comfortably just on the chair's handles. Subjects were instructed to carry on doing the activity while experiencing some mild symptoms but to cease if their suffering got severe or incapacitating. Subjects were permitted to increase the resistance by utilizing hand weights if they could complete the exercises without experiencing minimal physical discomfort. Table 1 describes the Powerball workout protocol that will be used and performed (Figure 5).

**Table 1:** Level of exercise values A: mild; B: moderate; C: severe

Weeks	Type of group	Exercise (set* time)	Name of training	Rest between sets	Rest between each exercise	Situation implementation of exercise	The total duration of the exercise
1^st^ week	A	Exercise 1: (3*30s)	Wrist flexion	1 min	2 min	Sitting on the table	20 mins
	A	Exercise 2: (3*30s)	Wrist extension	1 min	2 min	Sitting on the table	
	A	Exercise 3: (3*30s)	Elbow flexion	1 min	2 min	Standing	
	A	Exercise 4: (3*30s)	Elbow extension	1 min	2 min	Standing (trunk flexion)	
2^nd ^week	B	Exercise 1: (4*45s)	Wrist flexion	1 min 30 sec	3 min	Sitting on the table	39 mins
	B	Exercise 2: (4*45s)	Wrist extension	1 min 30 sec	3 min	Sitting on the table	
	B	Exercise 3: (4*45s)	Elbow flexion	1 min 30 sec	3 min	Standing	
	B	Exercise 4: (4*45s)	Elbow extension	1 min 30 sec	3 min	Standing (trunk flexion)	
3^rd ^week	C	Exercise 1: (5*60s)	Wrist flexion	2 min	4 min	Sitting on the table	64 mins
	C	Exercise 2: (5*60s)	Wrist extension	2 min	4 min	Sitting on the table	
	C	Exercise 3: (5*60s)	Elbow flexion	2 min	4 min	Standing	
	C	Exercise 4: (5*60s)	Elbow extension	2 min	4 min	Standing (trunk flexion)	

Group B (Conventional Group)

**Figure 6:**
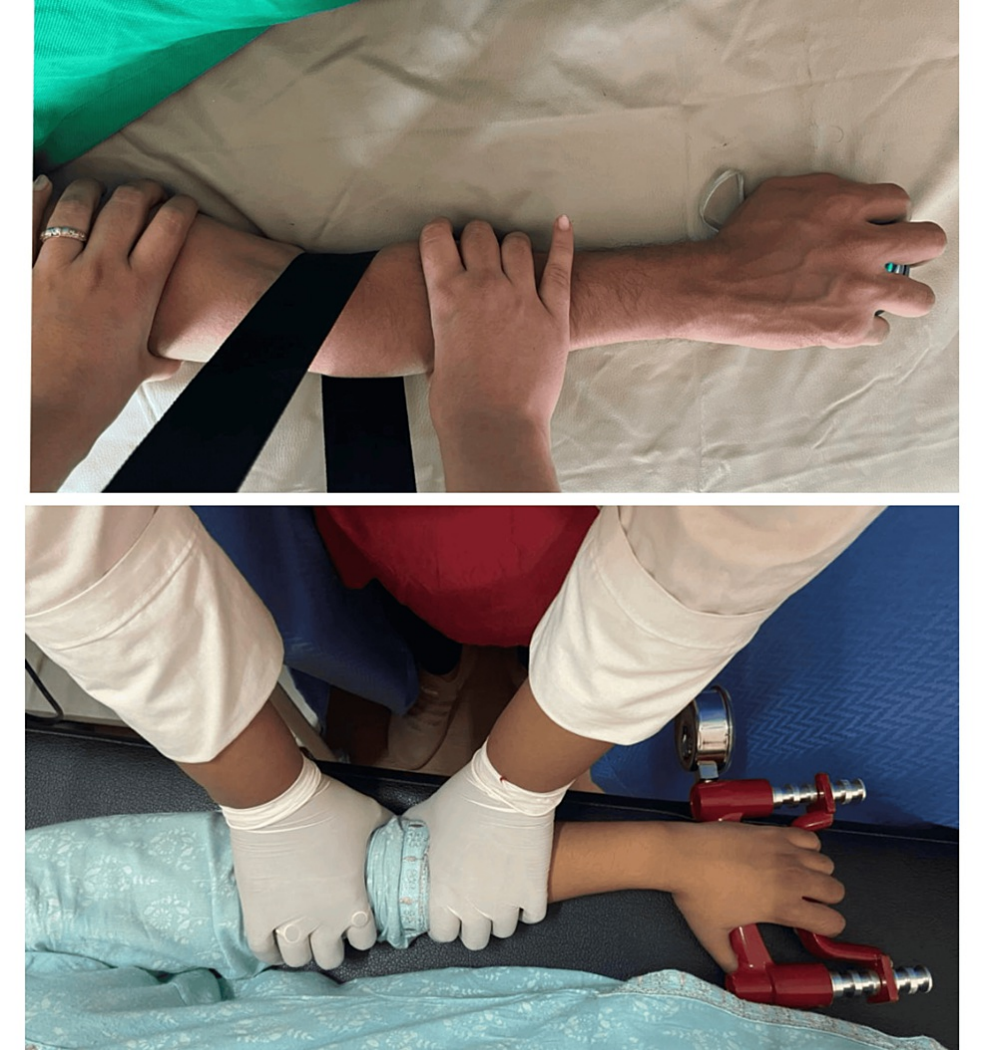
Showing the MMWM MMWM: Mulligan Mobilization with Movement

Group B underwent MMWM. It is indeed a method of manual treatment wherein the patient is resting flat with both the arm extended and the forearm in pronation, whereas continuous lateral glides are carried out in conjunction with the lateral epicondyle's physiological activity. The therapist stays at the participant's hand and fastens the belt over both their shoulders and the patient's forearm, just above the elbow. While doing glides, patients are instructed to create a fist or hold a dynamometer and stretch without feeling any discomfort. Throughout one visit, the physiotherapist carried out the process thirty times. A brief break period of one minute will be allowed after 10 repeats. Every treatment session lasts between 30 and 60 minutes. Over the next three weeks, there are scheduled five sessions each week, with three rounds of mobilizations with movement performed at each session. Table 2 describes the MMWM exercise protocol that was followed. Figure 6 shows the application and use of MMWM.

**Table 2:** Exercise protocol of MMWM MMWM: Mulligan Mobilization with Movement

Week	Exercise (sets*reps)	Rest between sets	Situation implementation of exercise	Total duration
1^st^ week (5 sessions)	(3*10)	1 min	Sitting on the table	32 mins
2^nd^ week (5 sessions)	(3*15)	1 min	Sitting on the table	47 mins
3^rd^ week (5 sessions)	(3*20)	1 min	Sitting on the table	62 mins

Outcome measures

Primary Outcome Measure

Includes grip strength, pain, and function post-intervention and VAS. PRTEE is a clinical outcome measure used to evaluate functional impairment and pain. There are two subscales in the index, one for pain and one for functional impairment. For patients with LE, it is the most accurate and dependable tool. The pain subscale consists of a total of 5 items, where 0 is no pain and 10 is the worst imaginable pain. The function subscale includes two components: specific activities consisting of a total of six items, and usual activities consisting of four items. Here, 0 means no difficulty, and 10 means unable to do. A hand dynamometer is a unique clinical piece of equipment used to test grip strength to identify and compute the hand and forearm muscles' maximum intensive contractile qualities. When the hand is utilized for flipping or lifting during a sport, such as baseball or tennis, dynamometers are used to assess grip strength. The VAS is a technique used to evaluate a trait or behavior that is thought to vary across a wide range of values and is difficult to calculate. In observational and clinical studies, it is commonly used to assess the degree or duration of discomfort when moving or at rest. The 10-cm pain scale is used to measure the level of pain felt during rest or exercise, with 0 representing no pain and 10 representing severe pain.

Secondary Outcome Measure

Includes range of motion (ROM), measured with a goniometer. The subjects will undergo evaluations while seated and when fully supported at the elbow in a supine posture. The forearm and arm must be exposed, and the individual must be free of any jewelry throughout the examination.

Statistical analysis

The chi-square test, Student's paired and unpaired t-tests, IBM SPSS Statistics for Windows, Version 27.0.1 (released 2020, IBM Corp., Armonk, New York, United States), and GraphPad Prism 7.0 (San Diego, USA) were used to perform descriptive and inferential statistics. The statistical analysis's level of significance was set at p<0.05. The statistical difference between Groups A and B was determined using Student's paired t-tests, and it was found to be significant.

## Results

The eligibility of each of the 88 patients that were enrolled was established. Due to 14 patients' refusals to participate, 19 patients' non-compliance with inclusion criteria, and five patients' extra justifications, 38 patients were left out. Patients in Groups A and B ranged in age from 22 to 49 and 21 to 45 years old, on average, respectively. Groups A and B had eight and 11 individuals representing age groups 21 to 30; eight and nine individuals representing age groups 31 to 40; and nine and five individuals representing age groups 41 to 50. The gender distribution of the participants was 18 and seven in Group A and 14 and 11 in Group B, which corresponded to male and female participants. The patient ages of the two groups were found to differ statistically significantly (p-value=0.0013) using the chi-square test. Table 3 describes the subjects' initial characteristics.

**Table 3:** Baseline characteristics Age in both groups is shown as Mean ± SD and the male: female ratio is given in percentage. S: significant; NS: not significant; p-value<0.05 is considered as significant

Baseline characteristics	Group A	Group B	p-value
Age range	22-49 yrs	21 - 45 yrs	0.0013, S
Male	18 (72%)	14 (56%)	0.24, NS
Female	7 (28%)	11 (44%)	

Table 4 shows the significant value between groups following rehabilitation as well as the statistical analysis of the main outcome measures that were examined. This study found that the PowerBall gadget improved pain and functional impairment in LE patients; that is, Group A's improvement in end measure scores was higher than Group B's.

**Table 4:** Mean VAS at rest and on activity, PRTEE, and grip strength pre- and post-treatment of Groups A and B and inter-group analysis The mean ± SD of all the primary outcomes within and between the group is shown in the above table. VAS: Visual Analogue Scale; PRTEE: Patient Rated Tennis Elbow Evaluation; S: significant; NS: not significant; p-value<0.05 is considered as significant

Primary outcome measures		Group A		p-value	Group B		p-value	Mean difference (X±SD)		p-value
		Pre-treatment	Post-treatment		Pre-treatment	Post-treatment		Group A	Group B	
VAS	Rest	6.56 ± 0.87	2.60 ± 0.76	0.0003, S	6.20 ± 1.12	1.96 ± 0.68	0.005, S	4.6 ± 1.0	4.0 ± 1.0	0.01, S
	Activity	7.56 ± 0.77	4.32 ± 0.59	0.003, S	7.28 ± 1.06	3.80 ± 1.00	0.007, S	3.5 ± 0.9	3.2 ± 1.1	0.03, S
PRTEE		52.68 ± 7.22	31.28 ± 5.80	0.001, S	56.72 ± 5.94	30.64 ± 5.00	0.055 NS	26.08 ± 6.36	21.40 ± 4.81	0.005, S
Grip strength		18.4 ± 2.8	23.0 ± 1.6	0.03, S	19.2 ± 2.0	22.3 ± 1.6	0.001, S	4.62 ± 2.48	3.11 ± 1.05	0.007, S

Table 5 presents the statistical analysis of the measured secondary outcome measure and the statistically significant difference in value between groups following rehabilitation. A range of motion (ROM) comparison between two groups before and after therapy was discovered in this study in elbow and forearm muscles, given the mean and standard deviation (SD), where Group A shows a significant increase in ranges as compared to that of Group B.

**Table 5:** Mean ROM of elbow and forearm at pre- and post-treatment of Groups A and B and inter-group analysis The mean ± SD of elbow and forearm range within and between the group is shown in the above table. ROM: range of motion; S: significant; NS: not significant; p-value<0.05 is considered as significant

Secondary outcome measure (ROM of elbow and forearm)	Group A		p-value	Group B		p-value	Mean difference (X±SD)		p-value
	Pre-treatment	Post-treatment		Pre-treatment	Post-treatment		Group A	Group B	
Elbow flexion	110.0 ± 4.8	135 ± 4.6	<0.001, S	109.7 ± 3.4	131.6 ± 7.2	<0.001, S	4.62 ± 2.48	3.11 ± 1.05	0.034, S
Elbow extension	110.2 ± 4.8	135.4 ± 4.6	<0.001, S	109.7 ± 3.4	131.6 ± 7.2	<0.001, S	25.20 ± 2.94	21.88 ± 7.00	0.034, S
Supination	75.0 ± 2.8	82.2 ± 2.6	<0.001, S	75.4 ± 2.8	80.9 ± 2.1	0.04, S	7.24 ± 2.07	5.48 ± 2.10	<0.001, S
Pronation	67.8 ± 2.4	77.0 ± 1.7	<0.001, S	76.3 ± 3.3	83.7 ± 2.5	<0.001, S	9.12 ± 2.60	7.40 ± 1.61	<0.001, S

Table 6 shows the significant value between groups after rehabilitation and the statistical analysis of the assessed secondary outcome measure. This study discovered that when wrist muscle ranges of motion were examined between two distinct groups before and after therapy, Group A's ranges significantly increased relative to Group B's.

**Table 6:** Mean ROM of wrist muscles at pre-and post-treatment in Groups A and B and inter-group analysis The mean ± SD of wrist range within and between the group is shown in the above table. ROM: range of motion; S: significant; NS: not significant; p-value less than 0.05 is observed as significant

Secondary outcome measure (ROM of wrist)	Group A		p-value	Group B		p-value	Mean difference (X±SD)		p-value
	Pre-treatment	Post-treatment		Pre-treatment	Post-treatment		Group A	Group B	
Wrist flexion	59.8 ± 6.4	75.9 ± 4.3	<0.001, S	58.8 ± 3.1	73.0 ± 1.4	<0.001, S	16.04 ± 3.38	14.12 ± 2.54	0.028, S
Wrist extension	55.0 ± 3.1	67.4 ± 2.0	<0.001, S	55.5 ± 2.2	66.6 ± 1.8	<0.001, S	12.40 ± 2.71	11.04 ± 1.51	0.033, S
Radial deviation	12.4 ± 1.5	17.8 ± 1.2	<0.001, S	13.0 ± 1.1	17.4 ± 1.1	<0.001, S	5.36 ± 1.68	4.36 ± 1.38	0.026, S
Ulnar deviation	25.3 ± 2.3	33.6 ± 1.1	<0.001, S	25.7 ± 2.0	32.0 ± 2.0	<0.001, S	8.32 ± 2.714	6.56 ± 1.73	0.002, S

**Figure 7:**
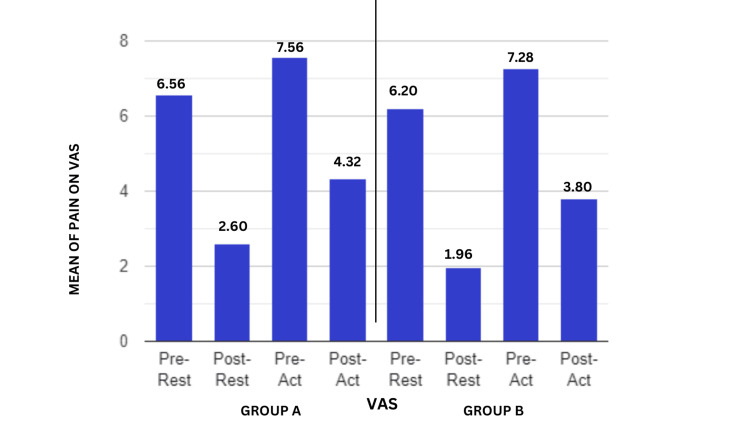
Comparison of VAS score in centimeters for pain in two groups at pre- and post-test VAS- Visual Analogue Scale; p-value<0.05 is considered as significant

Figure 7 shows the VAS scores for the activity and at rest before and after the therapy. The post-treatment results increased for both groups, although Group A's gains were more noticeable. When the intergroup analysis was conducted using Student's unpaired t-test, the VAS produced statistically significant results (t-value=2.10, p-value=0.041). This may indicate that the patients are experiencing less pain during and after their activities.

**Figure 8:**
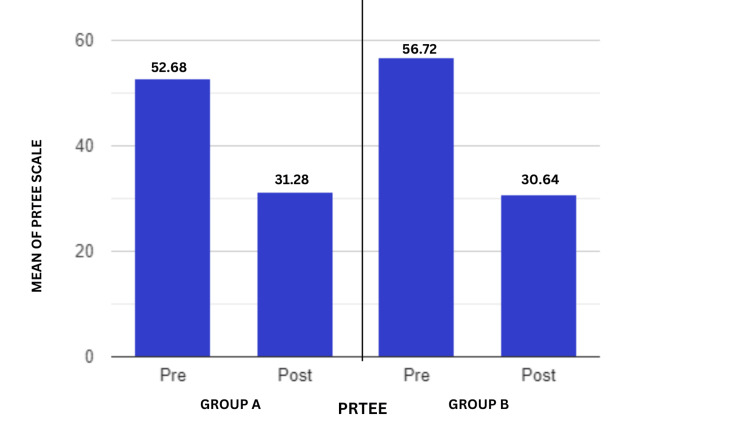
Comparison of the two groups pre- and post-test PRTEE Scale scores, which are computed out of 100 PRTEE: Patient-Rated Tennis Elbow Evaluation

Analogously, Figure 8 presents a comparison of the PRTEE scores before and after the intervention. Both groups had excellent outcomes, but Group A's was stronger, showing a reduction in the discomfort and functional impairment of the patient following treatment. The intergroup analysis was conducted using the student's unpaired t-test, and the PRTEE results revealed significant differences (t-value=2.93, p-value=0.005).

**Figure 9:**
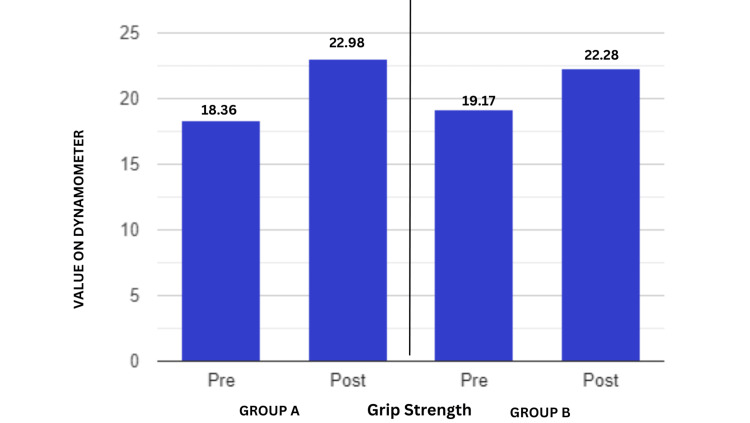
Comparison of grip strength measured in kilograms (kg) using dynamometers at pre- and post-test in two groups

Figure 9 demonstrates how the therapy plan improved the patients' grip strength after their recovery. This implied that the therapy also strengthened the muscles in the area. Student's unpaired t-test was employed for the intergroup analysis, and the grip strength results were significant (t-value=2.80, p-value=0.007).

**Figure 10:**
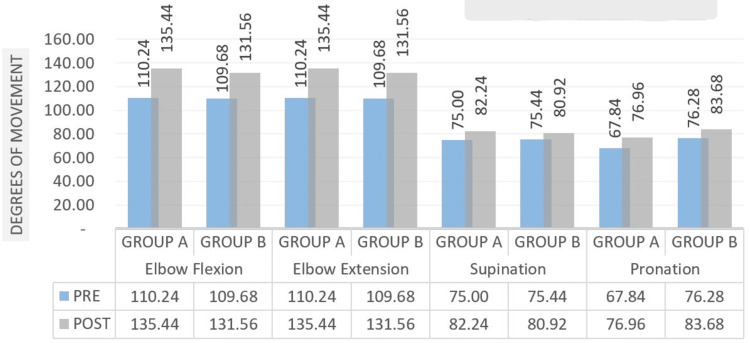
Comparison of ROM measured in degrees at pre- and post-test in two groups of elbow and forearm ROM: range of motion

Figure 10 offers the patient an improvement in range of motion following therapy. The results for elbow and forearm mobility were significant, and the Student's unpaired t-test was used for the intergroup analysis.

**Figure 11:**
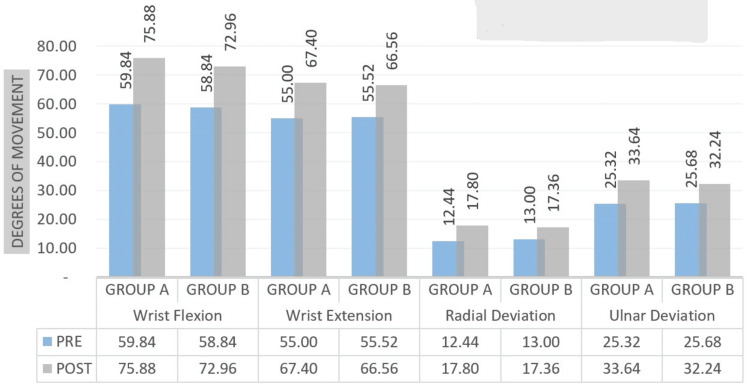
Comparison of ROM measured in degrees at pre- and post-test in two groups of wrist ROM: range of motion

Figure 11 shows the patient's range of motion improvement after rehabilitation. A Student's unpaired t-test was employed for the inter-group investigation, and the wrist movement showed statistically significant findings.

## Discussion

In this study, PowerBall and MMWM were compared to see how lateral epicondylitis patients' pain and function were impacted. Ultrasound and exercise significantly reduced pain and improved elbow function as measured by VAS and PRTEE, respectively; however, PowerBall exercises combined with therapeutic ultrasound and strengthening exercises are more effective in the treatment of LE.

In this study, we compared the VAS scores in the two groups before and after assessments while at rest and during activity. Additionally, both supervised PowerBall exercises and MMWM were successful in reducing pain, but PowerBall exercises accompanied by therapeutic ultrasound markedly improved the VAS score. The PRTEE scores were determined to be significant in both groups. Inferring from these numbers led to the conclusion that Group A was 21% more effective in lowering the PRTEE score and enhancing function.

The study covered LE, lateral tennis elbow etiology, and therapy. It is now believed to be associated with deterioration of the motor muscle system, alterations in the pain system, and local tendon sickness. Often, the diagnosis is made clinically using the symptoms, signs, and a traditional history [18]. The tendon of the ECRB has a very steady arterial blood flow [19]. This result suggests that probable hypovascular zones might exist at the tendons under the surface, resulting in degeneration and incomplete rupture and that this may be an etiologic factor for LE.

The conclusion from the study is that PowerBall wrist exercises can be helpful as management approaches for easing pain brought on by lateral epicondylitis and enhancing activities compromised by the tennis elbow [20]. Exercises that are eccentrically performed could ease symptoms of tendinopathy. PowerBall exercise significantly reduced pain, with a statistically higher reduction than in the MMWM group (in all or some of the parameters). In our study, we discovered that the supervised PowerBall group, which involved wrist extensor strengthening, was important in lowering pain on the VAS and raising the PRTEE Scale score, ultimately leading to an increase in functional improvement in everyday activities involving the elbow. Stretching and strengthening exercises performed in a clinic or physiotherapy session under supervision are more effective than workouts performed at home using a program [21].

Previous research backs up our findings of enhanced handgrip strength employing a "gyroscopic gadget" [22]. The results of this investigation, however, disagree with those of Balan et al. (2008) [23]. Increased muscle endurance did not statistically significantly affect hand grip strength, according to Balan et al. According to the research, strength may first improve as a result of neuromuscular adaptation (increased motor activation) [24], muscle hypertrophy [25], and probably a change in the kind of muscle fibers [26].

According to Bosco et al. (1999), the increase in muscle function following vibrating training is comparable to what happens after many weeks of intense resistance training [27]. In just three weeks, human skeletal muscle was able to adapt to reproduced hyper-gravity conditions, leading to an increase in leg extensor muscle behavior [28].

According to a 2010 report, the peculiarities of the illness, the diversity of pathophysiological pathways, the abundance of variables influencing the outcomes, and the absence of methodological concerns in the evidence currently available make it impossible to pinpoint an ideal approach to therapy for LE [29]. However, it is well known in clinical practice that MMWM techniques, particularly in LE patients, have a beneficial impact on treating pain within a short period [30]. Poor quality ratings of the pile of evidence for reported outcomes due to studies using a single session and limited sample numbers in mobilizing approaches trials, and shorter follow-up periods further prevent exact findings on the therapy of the condition. Although the method was created in the 1980s, it has just recently become more widely used [31].

Limitations

It is crucial to acknowledge the study's numerous flaws. Follow-up was not feasible because this was a time-limited study, indicating that the long-term impacts were not considered. The structured training program with pre-established exercises and the randomized controlled design are the greatest features of this study. The danger to internal validity was reduced because the same independent physiotherapist performed all of the examinations. Since a wider age range would have likely led to much more variation in the results, the small age range can be considered a strength.

## Conclusions

The study's findings indicate that a three-week program incorporating resistance training exercises mediated by a "gyroscopic device" enhances function, pain, grip strength, and the external rotators' concentric and eccentric strengths. The results of the post-test showed a significant difference between the two groups. Electromyography (EMG) recordings were omitted from the current investigation. Therefore, it is impossible to assess any neurogenic improvement immediately. Further study must be done using electromyography and motion analysis methods to assess changes in muscle and inter-joint coordination to better understand how control is altered. However, this study offers preliminary support for the use of resistance training exercises mediated via a "gyroscopic device" in tendinopathy sufferers to encourage improved function. This way, from the observations and results, we concluded that the PowerBall exercise is 21% more effective in the form of strengthening to improve pain and function on the PRTEE scale when exercise is paired with therapeutic ultrasound.

The study's conclusions demonstrate that strengthening the affected side, along with ultrasound and exercise, is beneficial for people with lateral epicondylitis. In the future, a comparable study might be carried out on patients of all ages in acute and chronic stages. The construction of a standard strengthening program for LE patients was also aided by this study, opening the door to several new kinds of research.
